# Cooked Rice Textural Properties and Starch Physicochemical Properties from New Hybrid Rice and Their Parents

**DOI:** 10.3390/foods13071035

**Published:** 2024-03-28

**Authors:** Yan Gao, Lin Zhang, Weiwei Chen, Weiyong Zhou, Guofu Deng, Gaoxing Dai, Jinsong Bao

**Affiliations:** 1 Key Laboratory of Nuclear Agricultural Sciences of Ministry of Agriculture and Zhejiang Province, Institute of Nuclear Agricultural Sciences, College of Agriculture and Biotechnology, Zhejiang University, Zijingang Campus, Hangzhou 310058, China; 2 Hainan Institute, Zhejiang University, Yazhou Bay Science and Technology City, Yazhou District, Sanya 572025, China; 3 Rice Research Institute, Guangxi Academy of Agricultural Sciences, Nanning 530007, China

**Keywords:** hybrid rice, starch, cooked rice texture, cooking and eating quality

## Abstract

Although great progress has been made in the development of hybrid rice with increased yield, challenges for the improvement of grain quality still remain. In this study, the textural properties of cooked rice and physicochemical characteristics of starch were investigated for 29 new hybrid rice derived from 5 sterile and 11 restorer rice lines. Except for one sterile line Te A (P1) with high apparent amylose content (AAC) (26.9%), all other parents exhibited a low AAC. Gui 263 demonstrated the highest AAC (20.6%) among the restorer lines, so the Te A/Gui 263 hybrid displayed the highest AAC (23.1%) among all the hybrid rice. The mean AAC was similar between sterile, restorer lines and hybrid rice. However, the mean hardness of cooked rice and gels of sterile lines were significantly higher than that of restorer lines and hybrid rice (*p* < 0.05). Pasting temperature and gelatinization temperatures were significantly higher in the hybrids than in the restorer lines (*p* < 0.05). Cluster analysis based on the physicochemical properties divided the parents and hybrid rice into two major groups. One group included P1 (Te A), P12 and P14 and three hybrid rice derived from P1, while the other group, including 39 rice varieties, could be further divided into three subgroups. AAC showed significant correlation with many parameters, including peak viscosity, hot peak viscosity, cold peak viscosity, breakdown, setback, onset temperature, peak temperature, conclusion temperature, enthalpy of gelatinization, gel hardness and cooked rice hardness (*p* < 0.05). Principal component analysis revealed that the first component, comprised of the AAC, peak viscosity, breakdown, setback, onset temperature, peak temperature, conclusion temperature and gel hardness, explained 44.1% of variance, suggesting AAC is the most important factor affecting the grain quality of hybrid rice. Overall, this study enables targeted improvements to key rice grain quality attributes, particularly AAC and textural properties, that will help to develop superior rice varieties.

## 1. Introduction

Rice (*Oryza sativa* L.) serves as a staple food source for most of the world’s population. The breeding of hybrid rice has considerably increased the yield of rice. Hybrid indica rice is the main rice type popularly grown in southern China, and the cultivation of hybrid rice with excellent eating quality is an important goal of rice breeding [1]. The cooking and eating quality (CEQ) is a quantitative trait with a complex mechanism of inheritance [2,3,4]. Compared with conventional indica rice, differences in the genetic background of the parents increase the uncertainty of CEQ of hybrid rice, making it more challenging to breeders [5,6]. With long-term breeding activities oriented towards good- quality hybrid rice development, many new good CEQ hybrid rice have been released [1]. However, the physicochemical properties and textural properties of the cooked rice have been less investigated, leading to the mechanism of good-quality hybrid rice being less understood.

Starch is the main storage substance of rice endosperm and is the principal edible component of rice [7], thereby acting as a key determinant of rice grain quality, especially CEQ [6,8,9]. The CEQ has been evaluated by physical and chemical properties, including apparent amylose content (AAC), gelatinization temperature (GT), gel consistency, and rapid visco analyser (RVA) viscosity properties [3,6,10]. AAC is one of the most important quality parameters associated with many physicochemical properties of rice [3]. High AAC rice has a firmer and less sticky texture following cooking than low AAC rice [3,11,12,13,14]. The RVA proves a useful tool to measure the pasting properties of starch in the food industry [15,16]. Previous studies have demonstrated a strong correlation between starch pasting properties and the eating quality of rice and food products, e.g., starch, cooked rice and rice noodle [10,16,17,18].

Rice is primarily consumed in a cooked form. The textural characteristics of cooked rice represent the most important qualities determining its CEQ and commercial value [19]. Wang et al. [19] indicated that the hardness of cooked rice was positively correlated with some aroma chemicals, such as E-2-hexenal, 2-hexanol-monomer, 1-propanol, and E-2-pentenal. In researching japonica rice, Xu et al. [20] reported that the overall sensory quality was negatively correlated with protein content and positively correlated with gel hardness, indicating that the protein content and gel hardness are important physicochemical properties for predicting the sensory quality of japonica rice.

The hybrid rice is different from conventional rice because the endosperm of hybrid rice originates from parent lines [1,6]. Therefore, the CEQ of hybrid rice is influenced by both parents [21]. Rare studies have been conducted to compare the CEQ between the sterile lines, restorer lines, and their hybrids. Comparison of the grain quality of hybrid rice and their parents may help to improve understanding of how the quality of hybrid rice was formed, and which parent contributed more to the hybrid rice.

The objectives of this study were as follows: (1) to compare the cooked rice textural properties and starch physicochemical properties of sterile lines, restorer lines, and their hybrids; (2) to understand the relationships between the textural properties and starch physicochemical properties; (3) to understand the quality relationship between hybrid rice and their parents. The results will provide reference for the improvement of hybrid rice quality.

## 2. Materials and Methods

### 2.1. Materials

A total of five sterile lines (A line), Te A (P1), Wantai A (P2), Xiang 117A (P3), Dingxiang A (P4) and Xiang 121A (P5), were selected for mating with eleven restorer lines (R), including Gui726 (P6), Gui965 (P7), Gui265 (P8), Gui559 (P9), Gui251 (P10), Gui471 (P11), Gui263 (P12), Gui516 (P13), Gui518 (P14), Gui685 (P15), and Meizhan (P16), forming an incomplete diallel cross, resulting in a total of 29 hybrid rice (Table 1). The hybrid rice and their parents were planted in the farm of Guangxi Academy of Agricultural Sciences in a randomized block with two replications. The corresponding maintainer lines (B line) replaced the A lines in the field experiment. The paddy rice was harvested in mid-November 2022. After storage at room temperature for two months, all the rice were milled to obtain polished rice. Polished rice was milled into rice flour using a UDY cyclone mill (UDY Corporation, Fort Collins, CO, USA). 

### 2.2. Apparent Amylose Content (AAC)

The iodine colorimetric method was used to determine AAC [22]. The absorbance of the resulting solution was measured at 620 nm using a microplate spectrophotometer (Epoch, BioTek, VT, USA). A standard curve was made from five standard rice samples with known amylose content, i.e., BP025 (17.0%), BP037 (28.5%), BP595 (2.0%), BP608 (8.0%), and SN05 (22.0%) [23]. A calibration curve was constructed with a coefficient of determination (R^2^) of 0.9903 (*p* < 0.0001).

### 2.3. RVA and Gel Textural Characteristics

The pasting viscosity of rice flour was measured using a rapid viscosity analyser (RVA, model 4500, Perten Instrument, Hägersten, Sweden) with the method described by Bao et al. [23]. A total of three grams of rice flour (12% moisture basis) and 25 g of double distilled water were weighed into aluminum cans, and was mixed and analyzed using the Rice program under the TCW3 (version 3.1, Thermocline for Windows software).

After RVA analysis, the aluminum canisters containing 12.5% moisture rice flour gel were sealed and stored in a refrigerator at 4 °C overnight. The textural properties were determined by a TA.XTC-18 texture tester (Shanghai Baosheng Industrial Development Co., Ltd., Shanghai, China), and the TPA programme was set to two cycles. The gel was compressed by 10 mm, with a probe diameter of 7 mm and a test speed of 1 mm/s. The textural parameters, including gel hardness (G-HD, g) and gel cohesiveness (G-COH), were read out by the software of the instrument.

### 2.4. DSC

The thermal (gelatinization) properties of starch were determined using a differential scanning calorimeter Q20 (TA Instruments, Newcastle, DE, USA) and the onset temperature (T_o_), peak temperature (T_p_), conclusion temperature (T_c_) and enthalpy (ΔH) were analyzed using Universal Analysis 2000 (Version 4.4A) software, according to the method described previously [23].

### 2.5. Texture Properties of Cooked Rice

A total of ten grams of polished rice samples were mixed with water with a rice-to-water ratio of 1:1.5 in a glass container with a lid and then allowed to stand for 30 min, and the glass container was then placed on the top layer of a Midea HEGON III Cooker (2100 W, Midea Life Electric Co., Ltd., Foshan, China) containing 2 L boiling water, and steamed for 40 min. After steaming, the heat was turned off, and the rice was kept warm for 20 min. A total of five grams of cooked rice was cooled to room temperature and placed in a plastic container with a diameter of 3.5 cm and a height of 1 cm. The textural properties were measured using a TA.XTC-18 texture tester (Shanghai Baosheng Industrial Development Co., Ltd.). The probe, which has a 15 mm diameter, was used and returned at a speed of 1 mm/s with a compression ratio of 50%. Measurements were repeated four times for each sample. The cooked rice hardness (C-HD), cooked rice cohesiveness (C-COH) and cooked rice chewiness (C-CHEW) were read out by the software of the instrument.

### 2.6. Statistical Analysis

All the samples were measured with two replicates. Analyses of variance (ANOVA) and correlation were conducted in SAS program version 8 (SAS Institute Inc., Cary, NC, USA). The least significant difference (LSD) multiple range test was conducted for comparing the means of samples at *p* < 0.05. Cluster analysis was conducted in SPSS statistics ver 20, employing hierarchical cluster analysis. (IBM Corp, Armonk, NY, USA). The method used was linkage between groups. The distances between samples were calculated using square Euclidean distances [24]. Additionally, principal components analysis (PCA) was performed in Origin 2021 [25].

## 3. Results

### 3.1. Apparent Amylose Content (AAC)

All the measured physicochemical properties and cooked rice textural properties are shown in Table S1, with the most important properties shown in Table 1. Among the sterile lines, the one with the lowest AAC was P5 (Xiang121 A, 11.6%), and its hybrid rice (P5/P8) had the lowest AAC among all the hybrid combinations (Table 1, Tables S1 and S2). The highest AAC among the sterile lines was found in the P1 (Te A, 26.9%), and the three hybrid rice derived from P1 (P1/P12, P1/P14, P1/P15) had AAC values of 23.1%, 21.9% and 22.1%, respectively (Table 1). Among the restorer lines, the one with the lowest AAC was P8 (Gui 265, 11.7%) (Table 1 and Table S1), and the hybrid derived from P8 had AAC values of 15.9% (P2/P8), 16.1% (P3/P8), 13.2% (P5/P8), and 15.3% (P4/P8), respectively (Table 1). Among the restorer lines, the highest AAC was found in P12 (Gui 263, 20.6%). The AAC of hybrid rice derived from P12 was 17.6% (P3/P12), 15.9% (P4/P12) and 23.1% (P1/P12) (Table 1). 

**Figure 1:**
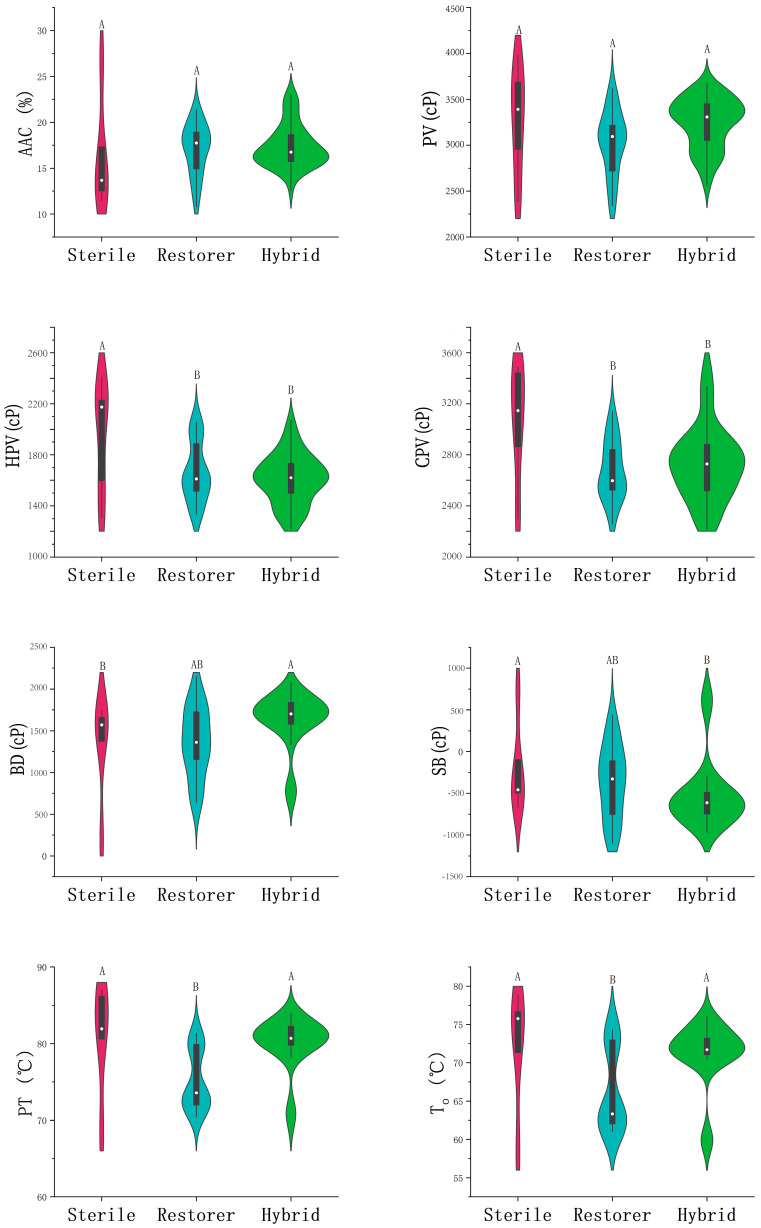

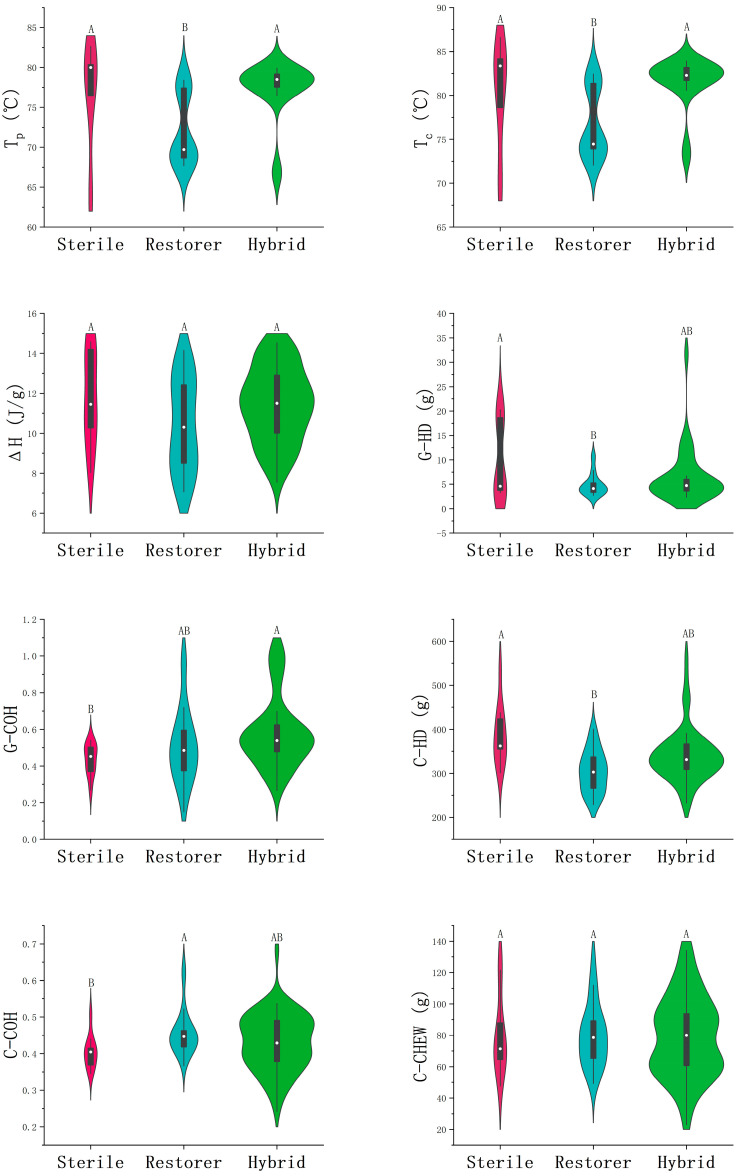
Distribution of mean values of physicochemical properties in male-sterile, restorer lines and their hybrid rice. Different letters above each violin plot indicate significant differences at *p* < 0.05. AAC: Apparent amylose content, PV: Peak viscosity, HPV: Hot paste viscosity, CPV: Cold paste viscosity, BD: Breakdown, SB: Setback, PT: Pasting temperature, T_o_: Onset temperature, T_p_: Peak temperature, T_c_: Conclusion temperature, ΔH: Enthalpy of gelatinization, G-HD: Gel hardness, G-COH: gel cohesiveness, C-HD: Cooked rice hardness, C-COH: Cooked rice cohesiveness, C-CHEW: Cooked rice chewiness.

The difference in AAC among the three groups is not significant, with mean AAC values of 16.3% for the sterile lines, 17.1% for the restorer lines, and 17.4% for the hybrid rice (Table S3; Figure 1).

### 3.2. Pasting Viscosity Properties 

The viscosity characteristics of three-line hybrid rice exhibit variations (Table 1 and Table S1, Figure 1). Among the sterile lines, P3 (Xiang117 A) displayed highest peak viscosity (PV, 3948 cP) and hot paste viscosity (HPV, 2377 cP), and its cold paste viscosity (CPV, 3458 cP) was almost the same as that of P5 (3462 cP). Its breakdown (BD, 1571 cP) was lower than P2 (Wangtai A) and P4 (Dingxiang A) (*p* < 0.05). A total of four sterile lines (P2–P5) exhibited a negative setback (SB), whereas P1 had a positive SB of 746 cP, but its BD was lowest among all the rice (Table 1 and Table S1).

Among the restorer lines, the fluctuations in PV, HPV, CPV, BD, SB, and pasting temperature (PT) were observed within the ranges of 2347–3560 cP, 1356–2033 cP, 2299–3056 cP, 657–2029 cP, −1034–296 cP and 70.8–80.3 °C, respectively (Table 1 and Table S2; Figure 1). The highest PV and BD and lowest SB was found in P13 (Gui516). P11, P12 and P14 were characterized by a relatively lower BD but a higher SB, while other parents had a negative SB value (Table 1).

Among the hybrid rice, PV, HPV, CPV, BD, SB and PT varied in the ranges of 2598–3629 cP, 1239–2048 cP, 2265–3478 cP, 658–2029 cP, −879–702 cP and 69.5–83.9 °C, respectively (Table S2). Notably, the hybrid rice of P3/P10 exhibited the highest BD (2029 cP). P1/P14 exhibited the lowest BD (658 cP) but highest SB (702 cP) among the hybrid rice varieties (Table 1 and Table S2). The lowest SB was from hybrid rice of P4/P13 (−879 cP) (Table 1). The hybrid rice of P3/P13 had a lower PV than both parents (*p* < 0.05), while the BD and SB fell between the two parents. However, the PV, BD and SB of P4/P13 were all between two parents (Table 1).

Among the parents and their hybrid rice, the sterile lines exhibited significantly higher HPV and CPV, compared to the restorer lines and hybrid rice (*p* < 0.05) (Figure 1; Table S3). The hybrid rice had higher BD but lower SB than restorer lines and sterile lines (*p* < 0.05) (Figure 1; Table S3).

### 3.3. Thermal Properties

Among the sterile lines, P1 had the lowest of gelatinization temperatures, i.e., onset temperature (T_o_, 58.1 °C), peak temperature (T_p_, 63.8 °C), conclusion temperature (T_c_, 69.8 °C), and lowest enthalpy of gelatinization (ΔH, 8.5 J/g), while other four parents exhibited with intermediate or high GT varieties, with T_p_ higher than 76 °C (Table 1, Tables S1 and S2; Figure 1). Among the restorer lines, P6, P9-P12, P14 and P15 were considered low GT rice, while others were intermediate or high GT varieties. Among the hybrid rice, most of them exhibited intimidate or high GT, but those derived from both low GT parents, such as P1/P12, P1/P14 and P1/P15, had low GT, while all others had intermediate or high GT. The coefficient of variation (CV) for the thermal properties of the sterile lines was relatively high, with values of 11.4%, 9.7%, 7.9%, and 20.8% for T_o_, T_p_, T_c_, and ΔH, respectively (Table S2). From the given data, CV of T_o_, T_p_, T_c_, and ΔH of the restorer lines were 8.2%, 6.1%, 5.2% and 22.2%, respectively. This indicates that there are great differences in the performance of restorer lines in these traits. However, variation of T_o_, T_p_, T_c_ and ΔH of hybrid rice were relatively low, which were 5.9%, 4.8%, 3.6% and 16.2%, respectively (Table S2). This indicates that the diversity of these traits in hybrid rice was relatively low, and the difference and diversity were relatively small.

Among the parents and their hybrid rice, the restorer lines exhibited lower T_o_, T_p_ and T_c_ than the sterile lines and the hybrid rice (*p* < 0.05) (Figure 1; Table S3), but no difference was found in ΔH among them (Figure 1; Table S3).

### 3.4. Textural Properties of Starch Gel

In sterile lines, rice paste gel hardness (G-HD) ranged from 3.3 g (P5) to 19.7 g (P4), with a mean value of 10.0 g (Table 1 and Table S2). In restorer lines, G-HD ranged from 2.9 g (P8) to 10.7 g (P14), with a mean value of 4.9 g. In hybrid rice varieties, G-HD ranged from 2.4 g (P2/P10) to 31.7 g (P1/P15), with a mean value of 6.6 g (Table 1 and Table S2).

In the sterile lines, the variation of rice paste gel cohesiveness (G-COH) was not significant, with a coefficient of variation of 4.3% (Table S2), and the range of variation was 0.41 g (P4)–0.45 g (P2) (Table 1). In the restorer lines, the variation of G-COH ranged from 0.34 g (P11)–0.97 g (P14), with a coefficient of variation of 37.0% (Tables S1 and S2). The G-COH levels of the hybrid rice varieties were in between the parents, with a variation range of 0.28 g–1.03 g, and a coefficient of variation of 32.5%, where the hybrid P3/P10 had the smallest G-COH. The maximum G-COH of the hybrid rice came from P2/P14 (Table 1 and Table S1).

The G-HD of the sterile lines was higher than the value of restored lines (*p* < 0.05), whereas G-COH was lower in the sterile lines, compared to the hybrid rice (*p* < 0.05) (Figure 1; Table S3).

### 3.5. Textural Properties of Cooked Rice

The range of C-HD varied from 329.5 g (P5) to 488.0 g (P1) for the sterile lines, with a coefficient of variation of 17.3%, from 249.3 g (P13) to 394.3 g (P6) with a CV of 14.9% for the restorer lines; and from 236.4 g (P4/P12) to 544.3 g (P5/P6) for the hybrid lines, with a CV of 17.8% (Table 1 and Table S2).

The C-COH of the sterile lines varied from 0.35 (P2) to 0.46 (P1), that of the restorer lines ranged from 0.41 (Gui 726) to 0.56 (Gui 251), and that of the hybrids varied from 0.26 to 0.61 (Tables S1 and S2).

The variation of C-CHEW was substantial, ranging from 57.8 g (P5) to 124.1 g (P1) for the sterile lines, 56.0 g (P13) to 120.6 g (P10) for the restorer line, and from 26.6 g (P2/P14) to 133.6 g (P2/P6) for the hybrids (Tables S1 and S2).

There was no significant difference in C-CHEW among the three lines. The C-HD of the sterile lines was higher than the value of restorer lines (*p* < 0.05), whereas C-COH were lower in the sterile lines, compared to the hybrid rice (*p* < 0.05) (Figure 1; Table S3).

### 3.6. Correlation of Physicochemical Properties

Positive correlations were found between AAC and HPV, CPV, SB, G-HD and C-HD, while negative correlations were found among AAC and PV, BD, T_o_, T_p_, T_c_ and ΔH (*p* < 0.05) (Table 2). Thermal properties (T_o_, T_p_, T_c_) had a positive correlation with PV and BD, and had a negative correlation with HPV, CPV, SB, G-HD and C-HD (Table 2) (*p* < 0.05). G-HD showed positive correlations with SB, G-COH and C-HD, and a negative correlation with PV, BD, T_o_, T_p_, T_c_ and ΔH (*p* < 0.05) (Table 2). Additionally, C-HD showed positive correlation with HPV, CPV and SB, and negative correlation with BD and C-COH (*p* < 0.05) (Table 2).

### 3.7. Cluster Analysis

**Figure 2:**
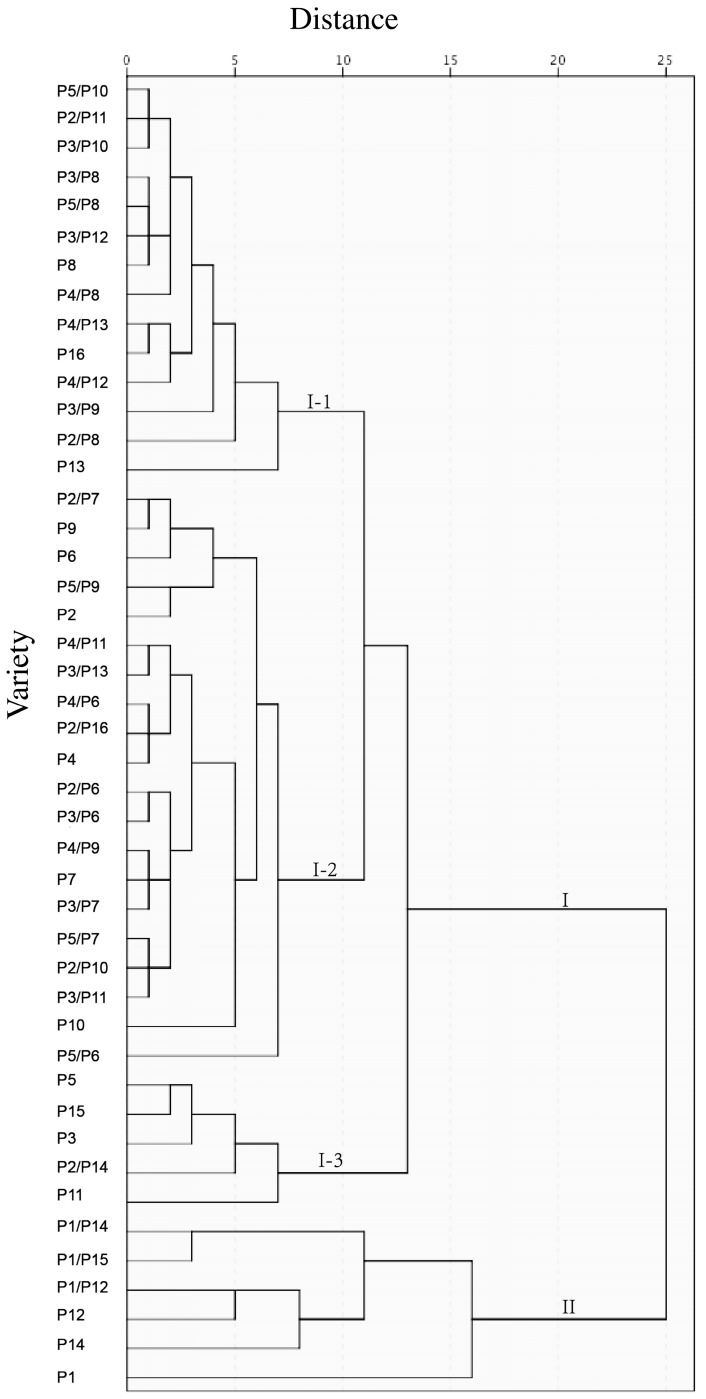
Cluster analysis of 45 rice varieties.

Cluster analysis showed that the 5 sterile lines, 11 restorer lines and 29 hybrid rice were generally classified into two major groups, of which the first group can be further divided into three categories, I-1, I-2, and I-3 (Figure 2).

There were four hybrids in I-1 with P8 (Gui 265) as the parent of restorer line, and they were classified in the same category, together with P8. Four hybrid rice and one parent of the sterile line (P3, Xiang117A) were likewise included in this category (Figure 2). In the I-2 group, three hybrids had P4 (Dingxiang A) as the parent of the sterile line, forming a distinct category along with P4. The first hybrid featured Wantai A (P2) as the parent of the sterile line, and four hybrids were grouped with P2. Furthermore, four hybrids, with Gui 726 (P6) as the parent of the restorer line, formed a separate cluster (Figure 2). The I-3 group consisted of two sterile lines, P3 and P5, two restorer lines. P11 and P15, and P2/P14 hybrid rice.

This cluster demonstrated a high AAC, with PV around 2700 cP, HPV around 2000 cP, CPV around 3300 cP, low BD (at about 700 cP), low PT (at around 70 °C), and low values for thermal properties. The G-HD exceeded 10 g, C-HD is also high, whilst C-CHEW remained low, at around 50 g (Figure 2).

**Table 2:** Correlation analysis of physicochemical properties of all rice accessions. * and ** indicate significance at *p* < 0.05 and *p* < 0.01, respectively.

	AAC	PV	HPV	CPV	BD	SB	PT	T_o_	T_p_	T_c_	ΔH	G-HD	G-COH	C-HD	C-COH
PV	−0.55 **														
HPV	0.31 **	0.07													
CPV	0.36 **	0.06	0.90 **												
BD	−0.65 **	0.78 **	−0.56 **	−0.51 **											
SB	0.67 **	−0.70 **	0.59 **	0.67 **	−0.95 **										
PT	−0.14	0.13	−0.01	0.03	0.12	−0.08									
T_o_	−0.68 **	0.68 **	−0.37 **	−0.34 **	0.80 **	−0.75 **	0.26 *								
T_p_	−0.64 **	0.69 **	−0.41 **	−0.36 **	0.83 **	−0.77 **	0.30 **	0.97 **							
T_c_	−0.60 **	0.65 **	−0.40 **	−0.34 **	0.79 **	−0.73 **	0.31 **	0.94 **	0.99 **						
ΔH	−0.40 **	0.29 **	−0.17	−0.14	0.35 **	−0.32 **	0.49 **	0.55 **	0.54 **	0.56 **					
G-HD	0.40 **	−0.49 **	0.17	0.20	−0.51 **	0.50 **	−0.13	−0.43 **	−0.45 **	−0.41 **	−0.23 *				
G-COH	−0.04	−0.14	−0.17	−0.16	−0.02	−0.01	−0.06	−0.08	−0.09	−0.09	0.07	0.50 **			
C-HD	0.33 **	−0.13	0.31 **	0.37 **	−0.31 **	0.37 **	0.12	−0.28 **	−0.28 **	−0.29 **	0.04	0.38 **	0.17		
C-COH	−0.02	0.05	−0.11	−0.25 *	0.11	−0.21 *	0.02	0.08	0.07	0.07	−0.11	−0.28 **	−0.27 **	−0.42 **	
C-CHEW	0.14	0.00	0.03	−0.07	−0.02	−0.06	0.12	−0.04	−0.05	−0.07	−0.04	−0.13	−0.19	0.15	0.80 **

AAC: Apparent amylose content, PV: Peak viscosity, HPV: Hot paste viscosity, CPV: Cold paste viscosity, BD: Breakdown, SB: Setback, PT: Pasting temperature, T_o_: Onset temperature, T_p_: Peak temperature, T_c_: Conclusion temperature, ΔH: Enthalpy of gelatinization, G-HD: Gel hardness, G-COH: Gel cohesiveness, C-HD: Cooked rice hardness, C-COH: Cooked rice cohesiveness, C-CHEW: Cooked rice chewiness.

### 3.8. Principal Component Analysis

**Figure 3:**
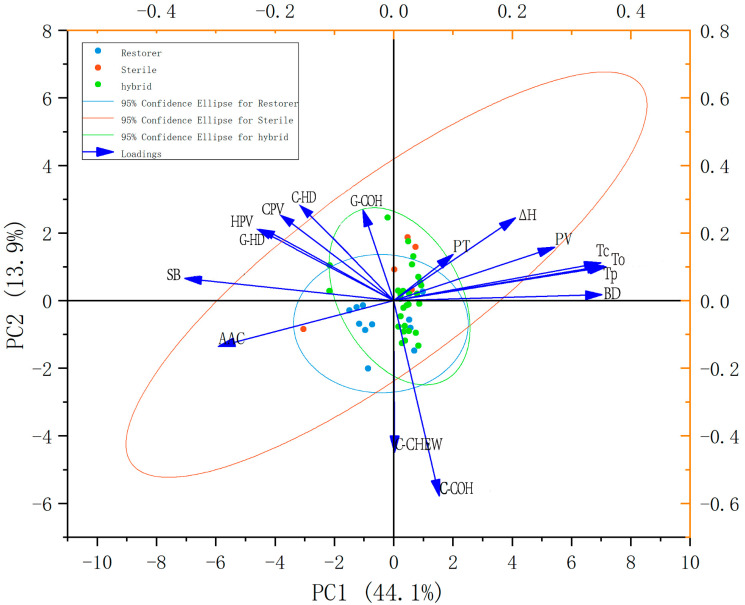
Principal component analysis of physicochemical properties of rice.

Figure 3 illustrates the results of the PCA of the 16 traits, and shows that the eigenvalues of the first four principal components exceeded one, and contributed a cumulative variance of 79.5% of the total variance (Table S4). This effectively summarized the essential information about the CEQ of rice varieties. The first principal component (PC1) exhibited a variance contribution rate of 44.1%. The PC1 comprised the AAC, PV, BD, SB, T_o_, T_p_, T_c_, and G-HD, which represented the AAC and main physicochemical characteristic factors of all rice varieties. The second principal component, which displayed a cumulative contribution of variance of 13.9%, was predominantly influenced by HPV and CPV. The third principal component, with a contribution of variance of 12.6%, highlighted G-COH, C-COH and C-CHEW as main factors. The fourth principal component, determined by PT, ΔH and C-HD, had a contribution of variance of 8.9% (Figure 3; Table S4).

## 4. Discussion

### 4.1. The Sterile Line, Restorer Line and Their Hybrids Have Different CEQ

With the development of hybrid rice technology, substantial progress in the improvement of rice yield has been achieved. However, the improvement of rice quality lags far behind rice yield, and has become a more important breeding aim in current breeding programs [3,6]. Rice grain quality encompasses basically four facets: appearance quality, milling quality, CEQ, and nutritional quality. Among them, CEQ is the most important trait because it affects market price and consumer acceptance of rice variety [3]. Rice CEQ can be evaluated by measuring the cooked rice textural properties and starch physicochemical properties.

The CEQ of sterile lines, restorer lines and their hybrids were compared in this study. The mean AAC between sterile lines, the restorer lines and their hybrid rice had no significant difference (Figure 1). Among most of the hybrid rice developed in the earlier period, the sterile male parent had a high AAC, resulting in the hybrid rice possessing a firm texture [2,26]. Due to continuous efforts in the breeding of the sterile rice, four sterile lines used in this study were characterized by low AAC, suggesting improvement of CEQ. Another sterile line (Te A, P1) with high AAC was still used in hybrid rice breeding because the hybrid rice derived from Te A was particularly used for rice noodle production. Zeng et al. [27] also indicated that among Southern China indica hybrid rice developed during the 2007–2017 period, a decreased AAC and increased gel consistency trend were found. From the results of AAC in this study, it is suggested that both parents, male and female, had improved their CEQ parameters. The hybrid rice had AAC ranging from 13.2% to 23.1%, but most of them were between 15% and 20% (Table 1), also suggesting that they have a good CEQ (Table 1).

Besides AAC, other starch physicochemical properties, such as RVA profiles, can also effectively predict CEQ. For rice with significant AAC, RVA can be easily predict the quality of their hybrid. For example, Xiang 117A (P3) had AAC of 13.2% and PV of 3948 cP, and Te A (P1) had AAC of 26.9% and PV of 2400 cP. When they mated with Gui263 (P12), P3/P12 showed low AAC (17.6%) and high PV (3576 cP), and P1/P12 showed high AAC (23.1%) and low PV (2846 cP), suggesting that P3/P12 had better CEQ than P1/P12. It has been suggested that RVA profiles can effectively differentiate the eating qualities of varieties with similar AAC [16,18]. Since all the restorer lines belonged to the low AAC class, their RVA profiles still differed largely, but most of them had the characteristic of large BD and small SB values. It was clear that both BD and SB differed significantly among the restorer lines (Table 1), suggesting that all the restorer lines still had different CEQ.

Difference in physicochemical properties reflects the difference in cooked rice texture [20]. Among the sterile lines, Te A has the highest G-HD (18.6 g) and G-HD (488 g), which can be attributed to Te A’s higher AAC (26.9%), as AAC always shows a positive correlation with the hardness of both starch gels and cooked rice (Table 2). Devi and Badwaik [28] found hardness was more elevated in the purple rice than other rice varieties, and attributed this elevation to the high AAC of purple rice variety. However, this is not always the case: for example, Xiang122A/Gui726 (P5/P6) has a high cooked rice hardness (544.3 g) but a low AAC (15.8%). Therefore, AAC cannot be the only predictor used to judge cooked rice hardness. In this study, since P5/P6 had a higher C-HD than that of P1, it is expected to have a lower CEQ. However, in the japonica rice with low AAC, the gel hardness, rather than AAC, showed a positive correlation with overall sensory quality. Since most parent and hybrid rice had a low AAC (Table 1), those with intermediate G-HD and C-HD might have a better CEQ. The hybrid rice of P5/P7, P4/P9, P2/P11, P3/P12 and P3/P13 had characteristics of G-HD between 5 and 7 g and C-HD between 300 and 320 g, which are expected to have a good CEQ. Further sensory quality tests of these hybrid rices are necessary to improve understanding of the relationship between G-HD, C-HD and sensory quality evaluated by panels.

### 4.2. The Relationships between the Starch Physicochemical Properties

Enquiries into the relationships between the starch physicochemical properties have been conducted in many studies [9,10,16,17,18], while the relationship between physicochemical properties and cooked rice texture is less understood. In this study, we found that AAC had a negative correlation with PV and BD, and had a positive correlation with HPV, CPV, SB, G-HD, and C-HD (Table 2). Zhu et al. [29] found in their research that AAC was negatively correlated with PV, BD and ΔH, and positively correlated with SB, which is consistent with our outcomes. We found that AAC was negatively correlated with T_o_, T_p_, T_c_ and ΔH (Table 2). Yao et al. [30] found that gelatinization temperature was negatively correlated with AAC, which is consistent with our findings. However, Zhu et al. [29] indicated that AAC had a positive correlation with T_o_, T_p_, and T_c_, which contrasted with our results (Table 2). Another study that used similar AAC rice as materials, conducted by Peng et al. [21], found that the gelatinization temperatures varied significantly, indicating no correlation between AAC and gelatinization temperature. Therefore, the discrepancy for the correlation between AAC and gelatinization temperature resulted from different genotypes used in the study. AAC has a positive correlation with G-HD and C-HD (Table 2), proving that high AAC rice generally has a firm texture [31]. Lin et al. [32] used Huajingxian 74 and its derived substitution lines as plant materials to analyze the textural properties of cooked rice, and found that C-COH exhibited an increasing tendency with the increase of AAC. However, in our study no association was found between AAC and C-COH (Table 2). Significant correlations were found for PV, HPV, CPV, BD, SB, T_o_, T_p_, T_c_, ∆H, G-HD, and C-HD and obtained from physicochemical tests (*p* < 0.05) (Table 2), which is consistent with previous studies [16,31,33]. PCA analysis indicated that the first component could explain 44.1% of the variance, which comprised the AAC, PV, BD, SB, T_o_, T_p_, T_c_ and G-HD. Since AAC had a significant correlation with these traits, it could be concluded that AAC is the major factor affecting the grain quality of hybrid rice.

### 4.3. The Quality Relationship between Hybrid Rice and Their Parents

The CEQ of hybrid rice is affected by both maternal and paternal parents [34]. Testing the cooking quality of their parents can aid in obtaining hybrid rice with better eating quality, thus improving the efficiency of hybrid rice quality breeding [35]. The analysis of physicochemical properties between the hybrids and their parents revealed that most of the CEQ of the hybrids were influenced by both parents [36]. Significant differences between the sterile lines and the hybrids were observed in some quality traits, including HPV, CPV, BD, SB, G-HD, G-COH, C-HD, and C-COH. This suggests that the restorer lines influenced these traits to some extent in hybrids. Whereas the restorer lines showed significant differences in traits such as SB, T_o_, T_p_, T_c_, G-HD, G-COH, C-HD, and C-COH, suggesting that the sterile lines affected these traits in hybrid rice to some extent.

The relationship between hybrid rice and their parents could also be found from cluster analysis (Figure 2). Within the I-1 group, the restorer line P8 and the four hybrids derived from it were clustered together, indicating the physicochemical properties of these hybrid rice varieties were affected by the parent P8. Within the I-2 group, three hybrids derived from P4 were clustered with P4, and four hybrids derived from P6 were clustered with P6. These facts suggest P4 and P6 influenced their hybrids more than the other parent. However, there were exceptions: for example, their hybrids derived from P3 clustered in the I-1 group, but P3 was in the I-3 group. The relationships between the parents and their hybrids are complex. Genetic analysis of the physicochemical properties may provide more understanding of the relationships.

Future work may be focused on the dissection of the genetic contribution of each parent to their hybrid. Since only 29 hybrid rice were made between 5 sterile parents and 11 restorer lines, the question of how to predict the CEQ of other combinations that were not made in this study by using the existing results derived from these 29 combinations is another challenge. These results will definitely help hybrid rice breeders to select suitable parent to make hybrid lines and then accelerate a breeding program.

## 5. Conclusions

The present study tested the physicochemical properties and cooked rice textural properties of hybrid rice and their parents developed in Guangxi province, China, emphasizing the importance of AAC and textural properties in determining cooking and eating qualities. The sterile lines, restorer lines and their hybrid rice had similar mean AAC, but they showed significant differences in RVA pasting viscosity and thermal properties, indicating that the CEQ of the hybrid rice differed. Cluster analysis based on these physicochemical properties classified all rice varieties into two major groups. PCA revealed that AAC, PV, BD, SB, T_o_, T_p_, T_c_ and G-HD in the first component could explain 44.1% of variance. Taken together with the fact that these traits had significant correlation with AAC, it is suggested that AAC is the most important factor affecting the grain quality of hybrid rice. Taking AAC, G-HD and C-HD into consideration, the hybrid rice of P5/P7, P4/P9, P2/P11, P3/P12 and P3/P13 are expected to have a better CEQ. The results provide a basis for continued screening and breeding efforts to improve the hybrid rice quality.

## Figures and Tables

**Table 1:** Main physicochemical and textural properties of 5 maintainer, 11 restorer and 29 hybrid rice varieties.

	Code	Name	AAC (%)	PV (cP)	BD (cP)	SB (cP)	T_p_ (°C)	ΔH (J/g)	G-HD (g)	C-HD (g)
Sterile line (n = 5)	P1	Te A	26.9 ± 1.1 a	2400 ± 15 e	186 ± 2 d	746 ± 40 a	63.8 ± 0.3 d	8.5 ± 0.3 c	18.6 ± 0.02 b	488 ± 49.7 a
	P2	Wangtai A	16.7 ± 0.6 b	3391 ± 72 c	1736 ± 15 a	−458 ± 13 c	76.5 ± 0.3 c	10.4 ± 0.3 b	4.6 ± 0.2 c	420.6 ± 3.8 ab
	P3	Xiang117 A	13.2 ± 0.5 c	3948 ± 35 a	1571 ± 0 b	−490 ± 5 c	82.2 ± 0.3 a	13.9 ± 0.3 a	3.8 ± 0.05 c	361.0 ± 5.5 c
	P4	Dingxiang A	13.1 ± 0.5 c	2960 ± 5 d	1643 ± 17 ab	−652 ± 2 d	80.2 ± 0.3 b	11.5 ± 0.3 b	19.7 ± 0.5 a	335.5 ± 19.3 c
	P5	Xiang121A	11.6 ± 0.2 c	3612 ± 73 b	1437 ± 65 c	−150 ± 12 b	79.9 ± 0.3 b	14.4 ± 0.3 a	3.3 ± 0.06 d	329.5 ± 28.5 c
Restorer line (n = 11)	P6	Gui726	16.2 ± 1.9 abc	3076 ± 14 c	1379 ± 33 c	−212 ± 37.5 cd	67.7 ± 0.0 g	7.8 ± 0.5 f	7.7 ± 0.2 b	394.3 ± 6.6 a
	P7	Gui965	16.1 ± 3.5 abc	3058 ± 80 c	1695 ± 75 b	−538 ± 12.5 e	76.7 ± 0.2 c	13.5 ± 0.03 a	3.0 ± 0.003 f	320.0 ± 17.6 bc
	P8	Gui265	11.7 ± 0.9 c	3445 ± 8 a	1827 ± 3 b	−962 ± 16.3 g	77.6 ± 0.2 b	13.8 ± 0.3 a	2.9 ± 0.2 f	299.0 ± 4.4 cd
	P9	Gui559	19.2 ± 0.3 ab	3067 ± 78 c	1441 ± 65 c	−308 ± 50.4 cd	69.8 ± 0.2 d	11.3 ± 0.5 c	4.1 ± 0.1 de	356.2 ± 7.9 b
	P10	Gui251	20.1 ± 1.2 a	3263 ± 46 b	1272 ± 52 cd	−426 ± 99 de	68.7 ± 0.1 f	9.1 ± 0.2 e	4.0 ± 0.04 e	310.2 ± 9.2 c
	P11	Gui471	16.9 ± 0.3 ab	3137 ± 26 bc	1191 ± 34 de	−120 ± 13.8 b	68.6 ± 0.0 f	9.0 ± 0.01 e	3.1 ± 0.2 f	245.0 ± 16.3 e
	P12	Gui263	20.6 ± 0.3 a	2689 ± 12 d	657 ± 13 f	296 ± 142 a	69.2 ± 0.08 e	10.3 ± 0.1 d	4.0 ± 0.5 e	328.8 ± 25.2 bc
	P13	Gui516	15.0 ± 0.3 bc	3560 ± 60 a	2029 ± 119 a	−1034.2 ± 63 g	78.3 ± 0.08 a	7.3 ± 0.2 f	4.8 ± 0.2 cd	249.3 ± 6.5 e
	P14	Gui518	16.3 ± 1.3 abc	2347 ± 2 e	792 ± 38 f	237 ± 20 a	68.7 ± 0.04 f	12.5 ± 0.1 b	10.7 ± 0.3 a	325.7 ± 0.8 bc
	P15	Gui685	17.9 ± 0.1 ab	2646 ± 72 d	1078 ± 97 e	−94 ± 82.8 b	69.8 ± 0.09 d	7.9 ± 0.6 f	5.6 ± 0.3 c	266.5 ± 0.7 de
	P16	Meizhan	17.7 ± 0.2 ab	3053 ± 46 c	1697 ± 27 b	−754 ± 3.8 f	77.9 ± 0.3 b	12.1 ± 0.3 bc	4.4 ± 0.5 de	254.3 ± 15.5 e
Hybrid rice (n = 29)	P2/P6	Wangtai A/Gui726	16.8 ± 0.3 e–i	3459 ± 35 cd	1730 ± 36 def	−628 ± 5 f–i	78.9 ± 0.6 def	10.2 ± 0.2 ij	3.5 ± 0.1 l–o	377.9 ± 0.1 d
	P3/P6	Xiang117 A/Gui726	16.3 ± 0.0 ghi	3275 ± 14 hi	1642 ± 11 f–i	−581 ± 2 fgh	78.7 ± 0.0 defg	11.1 ± 0.1 gh	3.7 ± 0.03 klmn	371.7 ± 4.7 de
	P5/P6	Xiang121A/Gui726	15.8 ± 0.6 ghi	3568 ± 8 ab	1898 ± 35 bc	−848 ± 11 jk	78.6 ± 0.3 fgh	14.3 ± 0.2 a	4.3 ± 0.3 jklm	544.3 ± 23.5 a
	P4/P6	Dingxiang A/Gui726	16.2 ± 0.2 ghi	3208 ± 4 ij	1727 ± 15 efg	−678 ± 4 hi	77.3 ± 0.2 klm	9.3 ± 0.1 klm	10.4 ± 0.3 d	351.3 ± 3.9 defg
	P2/P7	Wangtai A/Gui965	15.8 ± 0.7 ij	3290 ± 12 ghi	1525 ± 25 j	−323 ± 11 c	77.1 ± 0.1 lmn	12.2 ± 0.0 cdef	4.8 ± 0.07 ghij	362.7 ± 8.6 def
	P3/P7	Xiang117 A/Gui965	16.6 ± 0.5 f–i	3409 ± 28 def	1812 ± 24 cde	−602 ± 15 fgh	76.6 ± 0.2 n	11.4 ± 0.0 gh	5.3 ± 0.01 fghi	369.6 ± 2.2 de
	P5/P7	Xiang121A/Gui965	18.8 ± 0.1 cde	3300 ± 11 ghi	1703 ± 30 fg	−508 ± 12 def	76.7 ± 0.1 mn	11.8 ± 0.2 efg	5.5 ± 0.1 efgh	326.5 ± 19.3 f–i
	P2/P8	Wangtai A/Gui265	15.9 ± 0.6 ghi	3629 ± 24 a	1835 ± 85 cd	−825 ± 6 jk	79.3 ± 0.1 abcd	10.7 ± 0.1 hi	3.4 ± 0.1 mno	250.1 ± 17.7 kl
	P3/P8	Xiang117 A/Gui265	16.1 ± 0.3 ghi	3523 ± 18 bc	1874 ± 8 bc	−872 ± 17 k	79.1 ± 0.2 b–f	12.9 ± 0.5 bc	3.0 ± 0.01 nop	327.0 ± 9.3 f–i
	P5/P8	Xiang121A/Gui265	13.2 ± 0.7 k	3309 ± 28 gh	1843 ± 61 c	−830 ± 0 jk	79.3 ± 0.1 abcd	11.3 ± 0.4 gh	3.2 ± 0.02 nop	303.7 ± 16.1 hij
	P4/P8	Dingxiang A/Gui265	15.3 ± 0.0 jk	3049 ± 6 kl	1696 ± 14 fgh	−746 ± 3 ij	79.8 ± 0.0 a	12.0 ± 0.0 defg	2.6 ± 0.06 op	304.1 ± 5.2 hij
	P3/P9	Xiang117 A/Gui559	16.5 ± 1.6 f–i	3380 ± 75 d–g	1622 ± 75 g–j	−400 ± 1 cde	79.0 ± 0.2 cdef	13.1 ± 0.1 bc	3.6 ± 0.08 lmn	291.7 ± 0.4 ij
	P5/P9	Xiang121A/Gui559	17.7 ± 0.1 d–h	2887 ± 30 mn	1548 ± 33 ij	−374 ± 1 c	78.6 ± 0.4 efg	12.8 ± 0.1 bcd	3.6 ± 0.06 lmn	330.0 ± 2.9 fgh
	P4/P9	Dingxiang A/Gui559	20.2 ± 0.2 bc	3130 ± 1 jk	1723 ± 17 efg	−606 ± 3 fgh	77.9 ± 0.4 ijk	11.7 ± 0.2 efg	5.4 ± 0.09 fgh	320.5 ± 5.8 ghi
	P2/P10	Wangtai A/Gui251	18.6 ± 1.1 c–f	3332 ± 15 fgh	1703 ± 63 fg	−574 ± 6 fgh	78.7 ± 0.1 efg	14.2 ± 0.1 a	2.4 ± 0.07 p	329.5 ± 4.4 fgh
	P3/P10	Xiang117 A/Gui251	17.0 ± 2.4 e–i	3629 ± 44 a	2029 ± 60 a	−680 ± 19 hi	79.2 ± 0.1 bcde	13.2 ± 0.1 b	4.4 ± 0.2 ijkl	326.0 ± 26.3 f–i
	P5/P10	Xiang121A/Gui251	16.7 ± 1.0 e–i	3451 ± 37 cd	1830 ± 20 cde	−615 ± 80 fgh	79.0 ± 0.4 cdef	10.1 ± 0.4 ijk	2.6 ± 0.05 op	321.4 ± 7.7 ghi
	P2/P11	Wangtai A/Gui471	17.8 ± 0.8 d–h	3354 ± 14 efgh	1729 ± 50 d–g	−614 ± 5 fgh	77.8 ± 0.0 ijk	9.1 ± 0.2 lmn	5.4 ± 0.02 fgh	309.7 ± 1.2 hij
	P3/P11	Xiang117 A/Gui471	17.4 ± 0.7 e–i	3414 ± 21 def	1625 ± 53 f–j	−522 ± 22 efg	78.3 ± 0.2 ghi	7.9 ± 0.3 o	4.6 ± 0.07 hijk	331.9 ± 17.2 fgh
	P4/P11	Dingxiang A/Gui471	19.7 ± 1.2 cd	2925 ± 18 mn	1593 ± 20 hij	−660 ± 45 hi	77.6 ± 0.0 jkl	10.5 ± 0.1 hi	4.7 ± 0.2 hij	331.9 ± 11.7 fgh
	P3/P12	Xiang117 A/Gui263	17.6 ± 0.8 d–h	3576 ± 16 ab	1957 ± 9 ab	−860 ± 0.5 jk	79.1 ± 0.3 b–f	11.5 ± 0.5 fg	5.7 ± 0.1 efg	314.7 ± 31.4 ghi
	P4/P12	Dingxiang A/Gui263	15.9 ± 0.2 ghi	2971 ± 97 lm	1660 ± 15 fgh	−642 ± 15 ghi	78.0 ± 0.0 hij	9.4 ± 0.3 jklm	6.4 ± 0.4 e	236.4 ± 1.7 l
	P1/P12	Te A/Gui263	23.1 ± 0.1 a	2846 ± 35 n	799 ± 6.1 l	632 ± 21 a	66.4 ± 0.1 p	8.3 ± 0.3 no	12.7 ± 0.2 c	340.0 ± 7.5 efgh
	P3/P13	Xiang117 A/Gui516	15.8 ± 1.2 ij	2898 ± 12 mn	1658 ± 7 fgh	−596 ± 9.6 fgh	78.9 ± 0.2 defg	14.3 ± 0.1 a	6.1 ± 0.2 ef	319.0 ± 10.8 ghi
	P4/P13	Dingxiang A/Gui516	15.8 ± 0.1 ij	3425 ± 72 de	1875 ± 98 bc	−879 ± 8 k	79.5 ± 0.1 abc	12.9 ± 1.1 bc	12.5 ± 1.7 c	277.5 ± 9.9 jk
	P2/P14	Wangtai A/Gui518	18.0 ± 1.0 d–g	3096 ± 34 k	1336 ± 7 k	−395 ± 90 cd	77.7 ± 0.1 ijk	12.5 ± 0.4 bcde	14.6 ± 0.06 b	331.3 ± 1.0 fgh
	P1/P14	TeA/Gui518	21.9 ± 0.3 ab	2598 ± 41 p	658 ± 11 m	702 ± 5 a	67.6 ± 0.0 o	8.7 ± 0.1 mn	11.1 ± 0.7 d	428.6 ± 36.6 c
	P1/P15	TeA/Gui685	22.1 ± 0.3 ab	2743 ± 36 o	854 ± 19.6 l	492 ± 31 b	66.5 ± 0.5 p	9.9 ± 0.5 ijkl	31.7 ± 0.7 a	468.6 ± 3.5 b
	P2/P16	Wangtai A/Meizhan	15.3 ± 0.3 jk	3404 ± 47 def	1896 ± 50 bc	−871 ± 21 k	79.7 ± 0.1 ab	14.2 ± 0.3 a	4.2 ± 0.01 jklm	377.1 ± 6.3 d

Different letters in the same column with the same kind of rice indicate significant differences (*p* < 0.05). AAC: Apparent amylose content, PV: Peak viscosity, BD: Breakdown, SB: Setback, Tp: Peak temperature, ΔH: Enthalpy of gelatinization, G-HD: Gel hardness, C-HD: Cooked rice hardness.

## Data Availability

The original contributions presented in the study are included in the article/Supplementary Material, further inquiries can be directed to the corresponding authors.

## Supplementary Materials

The following supporting information can be downloaded at: https://www.mdpi.com/article/10.3390/foods13071035/s1, Table S1: AAC, pasting properties, thermal properties and textural properties of 45 rice varieties; Table S2: Mean, extreme value and coefficient of variation of stile lines, restorer lines, and their hybrid rice varieties; Table S3: Difference in the mean data between sterile lines, restorer lines and hybrid rice; Table S4: Principal component analysis of test results of all rice accessions.
