# Supplementary material for: Cooked Rice Textural Properties and Starch Physicochemical Properties from New Hybrid Rice and Their Parents
Source: Foods. 2024 Mar 28;13(7):1035. doi: 10.3390/foods13071035 (PMC11011368; doi:10.3390/foods13071035)
Supplement: Supplementary file 1 [file foods-13-01035-s001.zip › foods-2899021-supplementary.pdf]

Table S1. AAC, pasting properties, thermal properties and textural properties of 45 rice varieties

	Code	Name	AAC（%）	PV（cP）	HPV（cP）	CPV（cP）	BD（cP）	SB(cP)	PT(°C)	To(°C)	T_p(°C)	T_c (°C)	ΔH(J/g)	G-HD(g)	G-COH	C-HD(g)	C-COH	C-CHEW
Maintainer line (n=5)	P1	Te A	26.9±1.1	2400±15	2214±13	3147±53	186±2	746±40	68.4±1.3	58.1±0.3	63.8±0.3	69.8±0.3	8.5±0.3	18.6±0.02	0.4±0.08	488±49.7	0.5±0.05	124.1±2.4
	P2	Wangtai A	16.7±0.6	3391±72	1655±56	2933±69	1736±15	-458±13	81.45±0.9	71.7±0.3	76.5±0.3	78.8±0.3	10.4±0.3	4.6±0.2	0.5±0.03	420.6±3.8	0.4±0.008	63.8±0.7
	P3	Xiang117 A	13.2±0.5	3948±35	2377±35	3458±30	1571±0	-490±5	87.0±0.0	78.5±0.3	82.2±0.3	85.7±0.3	13.9±0.3	3.8±0.05	0.4±0.04	361.0±5.5	0.4±0.01	82.5±5.6
	P4	Dingxiang A	13.1±0.5	2960±5	1318±12	2308±10	1643±17	-652±2	81.6±0.0	75.8±0.3	80.2±0.3	83.8±0.3	11.5±0.3	19.7±0.5	0.4±0.1	335.5±19.3	0.4±0.008	71.4±3.0
	P5	Xiang121A	11.6±0.2	3612±73	2175±9	3462±21	1437±65	-150±12	86.2±0.0	76.6±0.3	79.9±0.3	83.4±0.3	14.4±0.3	3.3±0.06	0.4±0.07	329.5±28.5	0.4±0.009	57.8±10.2
Restorer line (n=11)	P6	Gui726	16.2±1.9	3076±14	1697±19	2865±24	1379±33	-212±37.5	72.2±0.2	64.0±0.3	67.7±0.3	72.1±0.3	7.8±0.3	7.7±0.2	0.7±0.003	394.3±6.6	0.4±0.001	87.2±2.5
	P7	Gui965	16.1±3.5	3058±80	1363±6	252l±68	1695±75	-538±12.5	79.1±0.8	72.0±0.3	76.7±0.3	81.4±0.3	13.5±0.3	3.0±0..0	0.3±0.06	320.0±17.6	0.4±0.006	86.7±2.6
	P8	Gui265	11.7±0.9	3445±8	1618±11	2483±24	1827±3	-962±16.3	80.3±0.4	73.2±0.3	77.6±0.3	81.6±0.3	13.8±0.3	2.9±0.2	0.5±0.07	299.0±4.4	0.4±0.03	68.0±5.8
	P9	Gui559	19.2±0.3	3067±78	1626±13	2759±28	1441±65	-308±50.4	72.8±0.8	62.3±0.3	69.8±0.3	74.2±0.3	11.3±0.3	4.1±0.2	0.6±0.04	356.2±7.9	0.4±0.03	93.7±7.9
	P10	Gui251	20.1±1.2	3263±46	1992±6	2838±145	1272±52	-426±99	72.8±0.8	61.9±0.3	68.7±0.3	73.7±0.3	9.1±0.3	4.0±0.04	0.4±0.06	310.2±9.2	0.6±0.04	120.6±8.8

	P11	Gui471	18.4±0.3	3137±26	1972±60	3056±40	1191±34	-120±13.8	73.2±0.4	62.1±0.3	68.9±0.3	74.6±0.3	9.7±0.3	3.2±0.2	0.3±0.08	266.2±16.3	0.5±0.02	66.0±1.7
	P12	Gui263	20.6±0.3	2689±12	2033±25	2985±54	657±13	296±142	73.6±0.0	63.3±0.3	69.2±0.3	74.0±0.3	10.3±0.3	4.0±0.5	0.4±0.09	328.8±25.2	0.4±0.003	72.3±6.9
	P13	Gui516	15.0±0.3	3560±60	1531±59	2526±3	2029±119	-1034.2±63	74.8±0.2	74.3±0.3	78.3±0.3	81.7±0.3	7.3±0.3	4.8±0.2	0.6±0.05	249.3±6.5	0.4±0.007	56.0±0.8
	P14	Gui518	16.3±1.3	2347±2	1555±40	2584±21	792±38	237±20	71.6±0.4	61.8±0.3	68.7±0.3	73.9±0.3	12.5±0.3	10.7±0.3	1.0±0.0	325.7±0.8	0.4±0.013	82.0±4.0
	P15	Gui685	17.9±0.1	2646±72	1568±25	2553±11	1078±97	-94±82.8	70.8±0.4	63.2±0.3	69.8±0.3	74.2±0.3	7.9±0.3	5.6±0.3	0.4±0.07	266.5±0.7	0.4±0.042	58.1±8.8
	P16	Meizhan	17.7±0.2	3053±46	1356±19	2299±43	1697±27	-754±3.8	79.5±0.4	73.7±0.3	77.9±0.3	82.0±0.3	12.1±0.3	4.4±0.5	0.5±0.002	254.3±15.5	0.5±0.09	90.9±21.1
	P2/P6	Wangtai A/Gui726	16.8±0.3	3459±35	1729±1	2831±0.3	1730±36	-628±5	79.9±0.8	71.2±0.3	78.9±0.6	82.2±0.1	10.2±0.2	3.5±0.1	0.4±0.006	377.9±0.1	0.5±0.04	133.6±0.5
	P3/P6	Xiang117 A/Gui726	16.3±0.0	3275±14	1633±3	2694±1	1642±11	-581±2	81±0.4	71.1±0.2	78.72±0.0	82.2±0.4	11.1±0.1	3.7±0.03	0.4±0.07	371.7±4.7	0.5±0.02	114.1±1.1
	P5/P6	Xiang121A/Gui726	15.8±0.6	3568±8	1670±43	2719±32	1898±35	-848±11	81.9±0.4	72.0±0.3	78.6±0.3	82.1±0.2	14.3±0.2	4.3±0.3	0.7±0.001	544.3±23.5	0.3±0.04	79.0±9.9
Hybrid rice (n=29)	P4/P6	Dingxiang A/Gui726	16.2±0.2	3208±4	1481±19	2530±23	1727±15	-678±4	79.9±0.1	71.4±0.2	77.3±0.2	81.8±0.1	9.3±0.1	10.4±0.3	0.9±0.01	351.3±3.9	0.4±0.04	81.8±11.5
	P2/P7	Wangtai A/Gui965	15.8±0.7	3290±12	1765±13	2967±24	1525±25	-323±11	81.9±0.5	73.1±0.0	77.1±0.1	81.1±0.1	12.2±0.0	4.8±0.07	0.6±0.01	362.7±8.6	0.4±0.02	93.4±3.5
	P3/P7	Xiang117 A/Gui965	16.6±0.5	3409±28	1598±3	2807±18	1812±24	-602±15	80.7±0.0	72.5±0.2	76.6±0.2	80.6±0.0	11.4±0.0	5.3±0.01	0.6±0.03	369.6±2.2	0.5±0.02	106.5±6.9
	P5/P7	Xiang121A/Gui965	18.8±0.1	3300±11	1596±41	2792±29	1703±30	-508±12	80.7±0..0	72.5±0.0	76.7±0.1	81.0±0.3	11.8±0.2	5.5±0.1	0.6±0.02	326.5±19.3	0.5±0.02	98.3±6.3

	P2/P8	Wangtai A/Gui265	15.9±0.6	3629±24	1793±61	2804±55	1835±85	-825±6	83.1±0.8	74.7±0.2	79.3±0.1	83.1±0.1	10.7±0.1	3.4±0.1	0.5±0.02	250.1±17.7	0.6±0.08	111.9±21.0
	P3/P8	Xiang117 A/Gui265	16.1±0.3	3523±18	1650±25	2651±8	1874±8	-872±17	83.9±0..0	74.7±0.1	79.1±0.2	82.9±0.1	12.9±0.5	3.0±0.01	0.4±0.06	327.0±9.3	0.4±0.003	58.2±1.5
	P5/P8	Xiang121A/Gui265	13.2±0.7	3309±28	1466±33	2479±33	1843±61	-830±0	81.9±0.4	74.4±0.1	79.3±0.1	83.1±0.1	11.3±0.4	3.2±0.02	0.5±0.009	303.7±16.1	0.4±0.02	50.6±7.5
	P4/P8	Dingxiang A/Gui265	15.3±0.0	3049±6	1353±20	2303±17	1696±14	-746±3	83.1±0.0	75.9±0.2	79.8±0.0	83.6±0.0	12.0±0.0	2.6±0.06	0.5±0.08	304.1±5.2	0.4±0.02	65.6±4.1
	P3/P9	Xiang117 A/Gui559	16.5±1.6	3380±75	1758±0	2979±1	1622±75	-400±1	81.1±1.2	71±0.3	79.0±0.2	83.6±0.3	13.1±0.1	3.6±0.08	0.4±0.02	291.7±0.4	0.5±0.01	83.6±4.5
	P5/P9	Xiang121A/Gui559	17.7±0.1	2887±30	1339±3	2513±4	1548±33	-374±1	78.3±0.0	71.1±0.6	78.6±0.4	83.2±0.0	12.8±0.1	3.6±0.06	0.5±0.01	330.0±2.9	0.4±0.02	60.2±5.7
	P4/P9	Dingxiang A/Gui559	20.2±0.2	3130±1	1406±15	2523±13	1723±17	-606±3	79.1±0.8	70.9±0.3	77.9±0.4	82.6±0.4	11.7±0.2	5.4±0.09	0.4±0.06	320.5±5.8	0.5±0.005	92.7±1.2
	P2/P10	Wangtai A/Gui251	18.6±1.1	3332±15	1628±48	2758±41	1703±63	-574±6	81±0.3	71.4±0.2	78.7±0.1	83.1±0.1	14.2±0.1	2.4±0.07	0.4±0.02	329.5±4.4	0.4±0.0	83.4±2.5
	P3/P10	Xiang117 A/Gui251	17.0±2.4	3629±44	1600±17	2949±2	2029±60	-680±19	81.0±1.2	72.3±0.1	79.2±0.1	83.5±0.4	13.2±0.1	4.4±0.2	0.7±0.15	326.0±26.3	0.4±0.02	56.5±0.1
	P5/P10	Xiang121A/Gui251	16.7±1.0	3451±37	1621±17	2836±64	1830±20	-615±80	81.0±0.4	73.0±0.2	79.0±0.4	82.6±0.2	10.1±0.4	2.6±0.05	0.3±0.01	321.4±7.7	0.4±0.006	63.3±2.6
	P2/P11	Wangtai A/Gui471	17.8±0.8	3354±14	1625±36	2740±41	1729±50	-614±5	79.5±1.2	71.1±0.0	77.8±0.0	81.4±0.0	9.1±0.2	5.3±0.02	0.5±0.07	309.7±1.2	0.4±0.008	64.8±2.2
	P3/P11	Xiang117 A/Gui471	17.4±0.7	3414±21	1789±33	2892±11	1625±53	-522±22	81.1±0.4	71.3±0.8	78.3±0.2	82.2±0.0	7.9±0.3	4.6±0.07	0.4±0.03	331.9±17.2	0.5±0.02	100.6±14.1
	P4/P11	Dingxiang A/Gui471	19.7±1.2	2925±18	1333±3	2265±48	1593±20	-660±45	79.9±0.0	71.1±0.0	77.6±0.0	81.9±0.1	10.5±0.1	4.7±0.2	0.6±0.06	331.9±11.7	0.5±0.03	104.2±5.1
	P3/P12	Xiang117 A/Gui263	17.6±0.8	3576±16	1619±25	2716±25	1957±9	-860±0.5	81.1±0.4	71.9±0.3	79.1±0.3	82.9±0.8	11.5±0.5	5.7±0.1	0.6±0.02	314.7±31.4	0.3±0.005	45.7±3.7

	P4/P12	Dingxiang A/Gui263	15.9±0.2	2971±97	1311±81	2329±96	1660±15	-642±15	79.3±0.2	71.7±0.2	78.0±0.0	82.4±0.4	9.4±0.3	6.4±0.4	0.5±0.008	236.4±1.7	0.5±0.007	62.2±1.5
	P1/P12	Te A/Gui263	23.1±0.1	2846±35	2048±28	3478±8	799±6.1	632±21	70.8±0.4	59.8±0.0	66.4±0.1	72.8±0.2	8.3±0.3	12.7±0.2	0.5±0.003	340.0±7.5	0.4±0.01	62.6±0.9
	P3/P13	Xiang117 A/Gui516	15.8±1.2	2898±12	1239±18	2301±28	1658±7	-596±9.6	82.4±0.1	73.9±0.3	78.9±0.2	83.2±0.5	14.3±0.1	6.1±0.2	0.6±0.02	319.0±10.8	0.5±0.005	109.0±6.1
	P4/P13	Dingxiang A/Gui516	15.8±0.1	3425±72	1550±26	2546±18	1875±98	-879±8	83.9±0.1	74.6±0.4	79.5±0.1	83.3±0.0	12.9±1.1	12.5±1.7	1.0±0.03	277.5±9.9	0.5±0.01	84.0±0.8
	P2/P14	Wangtai A/Gui518	18.0±1.0	3096±34	1760±27	2701±63	1336±7	-395±90	80.7±0.9	70.9±0.1	77.7±0.1	82.0±0.2	12.5±0.4	14.6±0.06	1.0±0.02	331.3±1.0	0.3±0.02	26.6±3.7
	P1/P14	TeA/Gui518	21.9±0.3	2598±41	1940±31	3300±35	658±11	702±5	72.0±0.8	60.4±0.0	67.6±0.0	74.7±0.1	8.7±0.1	11.1±0.7	0.5±0.02	428.6±36.6	0.3±0.02	48.6±1.8
	P1/P15	TeA/Gui685	22.1±0.3	2743±36	1889±17	3235±48	854±19.6	492±31	69.5±1.0	59.5±0.3	66.5±0.5	73.2±0.5	9.9±0.5	31.7±0.7	1.0±0.02	468.6±3.5	0.3±0.007	58.3±2.7
	P2/P16	Wangtai A/Meizhan	15.3±0.3	3404±47	1508±3	2533±25	1896±50	-871±21	81.9±0.4	75.2±0.2	79.7±0.1	83.6±0.3	14.2±0.3	4.2±0.01	0.6±0.05	377.1±6.3	0.4±0.006	90.0±0.2

AAC, Apparent amylose content; PV, Peak viscosity; HPV, Hot paste viscosity; CPV, Cold paste viscosity; BD, Breakdown; SB, Setback ; PT, Pasting temperature; To, Onset temperature; Tp, Peak temperature; Tc, Conclusion temperature; ΔH, Enthalpy of gelatinization; G-HD, Gel hardness; G-COH, Gel cohesiveness; C-HD, Cooked rice hardness; C-COH, Cooked rice cohesiveness; C-Chew: cooked rice chewiness.

Table S2. Mean, extreme value and coefficient of variation of stile lines, restorer lines, and their hybrid rice varieties

		Mean value	Min	Max	SD	CV（%）
Sterile line（n=5）	AAC	16.3	11.6	26.9	6.2	38.1
	PV	3262	2400	3948	601	18.4
	HPV	1948	1318	2377	444	22.8
	CPV	3062	2308	3462	477	15.6
	BD	1315	186	1736	640	48.7
	SB	-201	-652	746	560	-278.7
	PT	80.9	81.5	87.0	7.5	9.2
	T_o	72.1	58.1	78.5	8.2	11.4
	T_p	76.5	63.8	82.2	7.4	9.7
	T_c	80.3	69.8	85.7	6.3	7.9
	△H	11.7	8.5	14.4	2.4	20.8
	G-HD	10.0	3.3	19.7	8.4	83.9
	G-COH	0.4	0.4	0.5	0.0	4.3
	C-HD	386.9	329.5	488.0	67.0	17.3
	C-COH	0.4	0.4	0.5	0.0	10.5
	C-CHEW	79.9	57.8	124.1	26.4	33.0
Restorer line(n=11)	AAC	17.1	11.7	20.6	2.5	14.8
	PV	3031	2347	3560	354	11.7
	HPV	1662	1356	2033	238	14.3
	CPV	2675	2299	3056	236	8.8
	BD	1369	657	2029	445	32.5
	SB	-356	-1034	296	463	-129.9

	PT	68.3	70.8	80.3	21.3	31.2
	T_o	66.4	71.7	74.3	5.4	8.2
	T_p	72.1	67.7	78.3	4.4	6.1
	T_c	76.7	78.8	82.0	4.0	5.2
	△H	10.4	7.3	13.8	2.3	22.2
	G-HD	4.9	2.9	10.7	2.3	47.5
	G-COH	0.5	0.3	1.0	0.2	37.0
	C-HD	304.5	245.0	394.3	45.3	14.9
	C-COH	0.5	0.4	0.6	0.1	11.4
	C-CHEW	80.0	56.0	120.6	18.7	23.4
**F1 hybrid rice (n=29)**	AAC	17.4	13.2	23.1	2.2	12.9
	PV	3244	2598	3629	278	8.6
	HPV	1610	1239	2048	195	12.1
	CPV	2730	2265	3478	292	10.7
	BD	1634	658	2029	332	20.3
	SB	-514	-879	702	419	-81.5
	PT	80.0	69.5	83.9	3.5	4.4
	T_o	71.2	59.5	75.9	4.2	5.9
	T_p	77.3	66.4	79.8	3.7	4.8
	T_c	81.6	72.8	83.6	2.9	3.6
	△H	11.4	7.9	14.3	1.9	16.2
	G-HD	6.6	2.4	31.7	5.8	88.5
	G-COH	0.6	0.3	1.0	0.2	32.5
	C-HD	340.5	236.4	544.3	60.6	17.8
	C-COH	0.4	0.3	0.6	0.1	17.7

C-CHEW	78.9	26.6	133.6	25.0	31.7

Table S3. Difference in the mean data between sterile lines, restorer lines and hybrid rice

	Sterile line（n=5)	Restorer line(n=11)	Hybrid rice（n=29)
AAC	16.3^{A}	17.1^{A}	17.4^{A}
PV	3262^{A}	3031^{A}	3244^{A}
HPV	1948^{A}	1662^{B}	1610^{B}
CPV	3062^{A}	2675^{B}	2730^{B}
BD	1315^{B}	1369^{AB}	1634^{A}
SB	-201^{A}	-356^{AB}	-514^{B}
PT	80.9^{A}	68.3^{B}	80.0^{A}
T_o	72.1^{A}	66.4^{B}	71.2^{A}
Tp	76.5^{A}	72.1^{B}	77.3^{A}
Tc	80.3^{A}	76.7^{B}	81.6^{A}
△H	11.7^{A}	10.4^{A}	11.4^{A}
G-HD	10.0^{A}	4.9^{B}	6.6^{AB}
G-COH	0.4^{B}	0.5^{AB}	0.6^{A}
C-HD	386.9^{A}	304.5^{B}	340.5^{AB}
C-COH	0.4^{B}	0.5^{A}	0.4^{AB}
C-CHEW	79.9^{A}	80.0^{A}	78.9^{A}

Different superscript letters in the same line indicate significant differences ($p < 0.05$)

Table S4. Principal component analysis of test results of all rice accessions

Parameters	PC1	PC2	PC3	PC4
AAC	-0.801	0.15	0.103	-0.013
PV	0.86	0.294	0.064	0.037
HPV	-0.266	0.901	-0.006	0.057
CPV	-0.249	0.916	-0.124	0.095
BD	0.883	-0.311	0.057	-0.004
SB	-0.841	0.418	-0.137	0.038
PT	0.227	0.044	0.151	0.735
T_0	0.926	-0.196	0.024	0.138
T_p	0.928	-0.216	0.029	0.155
T_c	0.901	-0.226	0.008	0.182
ΔH	0.522	-0.082	-0.07	0.622
G-HD	-0.609	-0.108	-0.46	0.243
G-COH	-0.221	-0.384	-0.565	0.277
C-HD	-0.352	0.314	-0.214	0.577
C-COH	0.02	-0.235	0.903	-0.074
C-CHEW	-0.155	-0.064	0.864	0.3
Variance contribution value	44.126	13.941	12.576	8.873
Cumulative contribution value	44.126	58.067	70.643	79.516
